# The economics of adaptations to evidence-based practices

**DOI:** 10.1186/s43058-022-00345-8

**Published:** 2022-09-24

**Authors:** Ramzi G. Salloum, Todd H. Wagner, Amanda M. Midboe, Sarah I. Daniels, Andrew Quanbeck, David A. Chambers

**Affiliations:** 1 Department of Health Outcomes and Biomedical Informatics, College of Medicine, University of Florida, 2004 Mowry Road, Room 2243, Gainesville, FL 32610 USA; 2 Palo Alto Health Economics Research Center, Department of Veterans Affairs, Menlo Park, CA USA; 3 Department of Surgery, School of Medicine, Stanford University, Stanford, CA USA; 4 Center for Innovation to Implementation, Palo Alto Health Care System, Department of Veterans Affairs, Menlo Park, CA USA; 5 Department of Family Medicine and Community Health, School of Medicine, University of Wisconsin, Madison, WI USA; 6 Division of Cancer Control and Population Sciences, National Cancer Institute, Rockville, MD USA

**Keywords:** Adaptation, Adaptive, Economics, Cost, Economic evaluation

## Abstract

**Background:**

Evidence-based practices (EBPs) are frequently adapted in response to the dynamic contexts in which they are implemented. Adaptation is defined as the degree to which an EBP is altered to fit the setting or to improve fit to local context and can be planned or unplanned. Although adaptations are common and necessary to maximizing the marginal impact of EBPs, little attention has been given to the economic consequences and how adaptations affect marginal costs.

**Discussion:**

In assessing the economic consequences of adaptation, one should consider its impact on core components, the planned adaptive periphery, and the unplanned adaptive periphery. Guided by implementation science frameworks, we examine how various economic evaluation approaches accommodate the influence of adaptations and discuss the pros and cons of these approaches. Using the Framework for Reporting Adaptations and Modifications to Evidence-based interventions (FRAME), mixed methods can elucidate the economic reasons driving the adaptations. Micro-costing approaches are applied in research that integrates the adaptation of EBPs at the planning stage using innovative, adaptive study designs. In contrast, evaluation of unplanned adaptation is subject to confounding and requires sensitivity analysis to address unobservable measures and other uncertainties. A case study is presented using the RE-AIM framework to illustrate the costing of adaptations. In addition to empirical approaches to evaluating adaptation, simulation modeling approaches can be used to overcome limited follow-up in implementation studies.

**Conclusions:**

As implementation science evolves to improve our understanding of the mechanisms and implications of adaptations, it is increasingly important to understand the economic implications of such adaptations, in addition to their impact on clinical effectiveness. Therefore, explicit consideration is warranted of how costs can be evaluated as outcomes of adaptations to the delivery of EBPs.

Contributions to the literature

- Adaptations are a reality of implementing evidence-based practices and have economic implications
- Economic implications should be considered in decisions to adapt evidence-based practices
- Current frameworks to describe adaptations and specify the conditions under which adaptations are made can be helpful in identifying their resource implications
- Economic evaluation tools can guide how and when to assess the economic impact of adaptations

## Introduction

Allocating resources to the implementation of interventions requires an understanding of the relationship between resources expended and health outcomes achieved by the program or intervention. Economic evaluation methods that have traditionally been applied to measure value and compare the costs and consequences of health interventions [1] can also inform whether strategies designed to improve the quality of health care delivery and the uptake of evidence-based practices (EBPs) represent a cost-effective use of limited resources. An increasing number of health economic evaluations are nested within implementation and improvement research studies [2]. These studies estimate the costs of delivering EBPs (intervention costs), the costs of the implementation strategies to enhance their delivery (implementation costs), and the downstream health care costs subsequent to the implemented EBP (downstream costs) [3].

Despite the growing use of economic evaluation in implementation science, little attention has been given to economic issues and to the methodological challenges of evaluating the marginal costs of implementation attributable to adaptations that often occur to EBPs during the implementation process [4]. This article offers a unique perspective on the economics of adaptation, an evolving area of the field, by discussing practical approaches to evaluate the economic consequences of adaptation (i.e., what is planned at baseline vs. what gets delivered). This topic is of increased importance to public health and health care delivery with the introduction of new approaches that integrate adaptive, contextually sensitive continuous quality improvement, particularly within learning health care systems [5]. Previous research suggests that adaptations are widespread [6, 7], underscoring the importance of understanding the effectiveness and economic implications of various adaptations to public health and health care delivery systems.

## Background

### Defining adaptation

There is an ongoing debate regarding what is meant by the term *adaptation* [8], and we use a definition that is broad but specific enough to advance the discussion of how to measure the economic impact of adaptations. Adaptation of EBPs has been defined as the process of “thoughtful and deliberate alteration to the design or delivery of an intervention,” with the goal of improving its fit or effectiveness in a new context [9]. The origins of adaptation can be traced to its roots in the Diffusion of Innovations theory [10], originally defined as “re-invention” and discussed as early as 1972 by Charters and Pellegrin [11]. Originally thought to be unambiguously negative, re-invention has increasingly been recognized as a fundamental part of the adoption and implementation process. The most common reasons for adaptation have included the need for a culturally appropriate program, a new target population, a new community or clinical setting, the desire to improve ease and feasibility of implementation, attempting to make the program more widely accessible, and condensing the original intervention [7]. Although adaptations can be made to the implementation strategy used to deliver EBPs into a new setting, this paper focuses on adaptations to EBPs which can occur even in the absence of implementation strategies.

### The role of economics in the science of adaptation

There is growing recognition that adaptation is a necessary step in optimizing EBP implementation and that it is essential to the uptake and sustainment of EBPs. There are common steps involved in adaptation processes, each of which may have economic implications. These steps typically include conducting an organizational assessment, determining the level of change, and consulting stakeholders or experts before adapting the intervention, preparing new materials, training staff members, implementing the adapted intervention, evaluating the adapted intervention, and determining needed changes based on action step assessments [7].

Given the adaptations may have resource implications, there is a need to advance the science at the intersection between adaptation and economic evaluation. Despite their widespread prevalence, adaptations are understudied, with knowledge gaps surrounding their context, the adaptation decision itself, the positive and negative outcomes associated with various levels of adaptation, and the robustness of adaptations across different contexts and types of interventions [6]. Early efforts have been directed at studying the impact of adaptation on individuals and organizations, including an initiative by the Robert Wood Johnson Foundation to systematically investigate the local adaptation of evidence-based practices [12]. Advancing our economic understanding of adaptation is consistent with calls to develop strategies to improve the science of adaptation in the context of implementation that would more comprehensively describe the needed fit between EBPs and their settings, and embrace opportunities for ongoing learning about optimal EBP delivery over time [13].

### Progression of economic evaluation in implementation research

Economic modelling can inform those planning to scale up EBPs. In this respect, various approaches to the economic evaluation for implementation of EBPs can be applied to study adaptations. When evaluating EBPs, estimates of the expected benefit for each patient can be summed up to estimate the net effect for a group of patients, which can be used to estimate the net effect for a health care system. Sculpher first proposed the evaluation of implementation strategies by estimating their cost-effectiveness ratio in 2000 [14]. This model assumes that the strategies under evaluation can achieve perfect implementation conditions with patterns of clinical practice remaining static. In 2001, Mason and colleagues suggested a framework for economic evaluation that examines the cost-effectiveness of implementation strategies that help to achieve the adoption of treatments in addition to the cost-effectiveness of the treatments [15]. This framework recognized that interventions do not achieve perfect implementation in the real world, and therefore, it is important to value the actual uptake of the intervention. However, costs on this framework are estimated at the patient level, which is less useful to decision-making that occurs at the program level. Additionally, while this framework acknowledges implementation gaps as a problem in economic modeling, it does not directly address adaptations and their consequences with respect to economic evaluation. Over the past two decades, analytic methods have been proposed to measure the expected value of the improved implementation of health care interventions at the patient level [16, 17]. However, the literature is scarce on approaches to directly address adaptation as a necessary step in optimizing implementation. In response, this paper discusses available frameworks and methods that can be applied to economic evaluation of adaptations, as well as the needed for new methods and approaches to advance the science of adaptation from an economic evaluation lens.

## Methodology

### Framework for characterizing the economic drivers and consequences of adaptations

Stirman et al. developed the Framework for Reporting Adaptations and Modifications to Evidence-based interventions (FRAME) for describing and measuring adaptations that organizations make to fit an evidence-based intervention to their setting [6]. The framework was based on a systematic review of adaptations to evidence-based interventions. The latest iteration of this framework [18] includes eight domains: (1) when and how in the implementation process the adaptation was made, (2) whether the adaptation was planned/proactive or unplanned/reactive, (3) who determined that the adaptation should be made, (4) what is adapted, (5) at what level of delivery the adaptation is made, (6) type or nature of context or content-level adaptations, (7) the extent to which the adaptation is fidelity-consistent, and (8) the reasons for the adaptation, including the intent or goal of the adaptation (e.g., improve fit, adapt to a different culture, reduce costs).

Across all domains of the framework, there are economic drivers and implications that should be considered. Costs can vary according to (1) the timing of the adaptation; (2) whether they are planned; (3) the decision-maker responsible for the adaptation; (4) whether the adaptation is to the EBP or implementation strategy; (5) whether the EBP is delivered at the patient, practice, or organizational level and the nature of the adaptation; (6) the nature of the content adaptation; (7) whether the adaptation preserves fidelity to the core components of the EBP; and (8) the reason or motivation for the adaptation. In some cases, economic factors might be driving the adaptation, and researchers may seek to understand the mechanisms by which costs motivate adaptations to a program and the role they play in influencing the likelihood of sustainability over time. Table 1 presents examples within each domain of the FRAME, along with its cost relevance, considerations for cost measurement, and comparisons of its implications according to whether the adaptation was planned or unplanned. For example, adaptation can focus on either the EBP or the implementation strategy. Delineating adaptation costs of the implementation strategy compared with the EBP is crucial for scaleup efforts. Variation in adaptation costs may be related to the complexity of the intervention to be implemented [19]. Potential strategies to reduce the cost of adaptations may include starting with smaller adaptations and building upon successful experiences and sharing learnings from adaptations early and often through “train-the-trainer” or communities of practice approaches.

**Table 1:** Adaptation implications and considerations for cost measurement, organized by the Framework for Reporting Adaptations and Modifications (FRAME) [6]

Domain/construct	Example	Cost relevance/implications	Considerations for cost measurement	Planned vs. unplanned
1. Timing	Adaptation occurs in the pre-implementation or planning phase vs. implementation phase	Early-phase adaptations, especially prior to implementation, may be less costly than late-phase adaptations	Time of initiation, frequency, and duration period of adaptation. These may vary across sites due to capacity	More difficult to capture time variables in unplanned adaptations
2. Planning level	Unplanned/reactive vs. planned/proactive	Unplanned adaptations may require additional resources not originally budgeted for. Alternatively, unplanned adaptations may reduce costs when they are aimed at improving efficiency during the implementation process	Appropriate data capture strategy is imperative for assessing continual iteration on the adaptation to measure incremental cost on the margin (reactively or proactively)	Reporting system is already in place for measuring planned adaptations; reporting system for unplanned adaptation may be created impromptu
3. Decision-maker	Funder or payer vs. other stakeholders (e.g., political leader, clinician, intervention recipient)	A funder/payer involved in the decision to adapt may be more cost-conscious than another stakeholder who is less affected by costs	Estimated cost impact of adaptation may be contrary to what was anticipated	Projected changes in cost due to planned adaptations could be estimated in BIA a priori for funder buy-in to keep/change decision
4. Adaptation focus	Adaptation to the EBP vs. implementation strategy	Adapting complex implementation strategies may be more costly than adapting less-complex strategies	Delineating adaptation costs of implementation vs. EBP is crucial for extrapolation/scaling. Variation in adaptation costs may be related to the complexity of the intervention	Projected changes in cost due to planned adaptations to implementation vs. EBP could be estimated a priori for budgeting purposes
5. Delivery level	Individual patient/participant vs. clinic/unit level vs. organization/health system	Adaptations at the individual patient level may have a low unit cost but lead to high total cost when extend to a patient population, whereas higher level adaptations may involve costly adaptations to infrastructure	Depending on the delivery level; new data collection on direct, both fixed and variable, and indirect costs may be needed to assess adaptation costs. Effects on unit cost may be more difficult to interpret at higher levels	Projected changes in cost due to planned adaptations in delivery level could be estimated a priori for budgeting
6. Nature of content adaptation	Tailoring or refining, adding or removing elements, shortening or lengthening	Extending the intervention or implementation strategy may result in higher cost whereas condensing it may result in lower cost	May require mixed methods to collect all data necessary for accurately discerning the nature of the adaptation	Need for both quantitative and qualitative data may be particularly acute for unplanned adaptations where context is not well-known
7. Relationship to fidelity	Adaptation that preserves core elements vs. one that fails to do so	Departure from core elements may increase cost if additional element(s) are needed or reduce cost if certain element(s) are no longer needed when implementing in a new context	May require mixed methods to collect all data necessary for accurately discerning the nature of the adaptation	May need to consider loss of more sites, providers, and/or patients with unplanned adaptation
8. Reason or motivation	Increase reach or engagement, improve fit, reduce cost	Motivations for the adaptation (i.e., increasing positive outcomes) may also increase cost. Determining when, why, and how reduced costs align with other motivations should be addressed at the outset	Estimated cost impact of adaptation may be contrary to what was anticipated	Projected changes in cost due to planned adaptations could be estimated a priori to support or refute reasoning for adaptation

#### Mixed methods economic evaluation

Guided by the FRAME and other tools [20], mixed methods approaches can be highly useful to elucidate the economic reasons driving the adaptations to improve the generalizability of findings, as well as their economic consequences [21]. Mixed methods can also inform sensitivity analysis that is often a critical component to test the assumptions made in economic evaluation [22], by further identifying and explaining variations in cost at different levels (e.g., provider, practice) and for various stakeholders. For implementation planning purposes, it is useful to understand the dimensions upon which planned adaptations may be warranted and to establish a range in costs associated with planned adaptations that are likely to occur. Whereas estimating the cost implications of adaptations can be useful, more nuanced economic evaluation than simply measuring costs should be carried out in early phases of implementation and improvement program planning.

### Addressing the economic consequences of adaptation

**Fig. 1:**
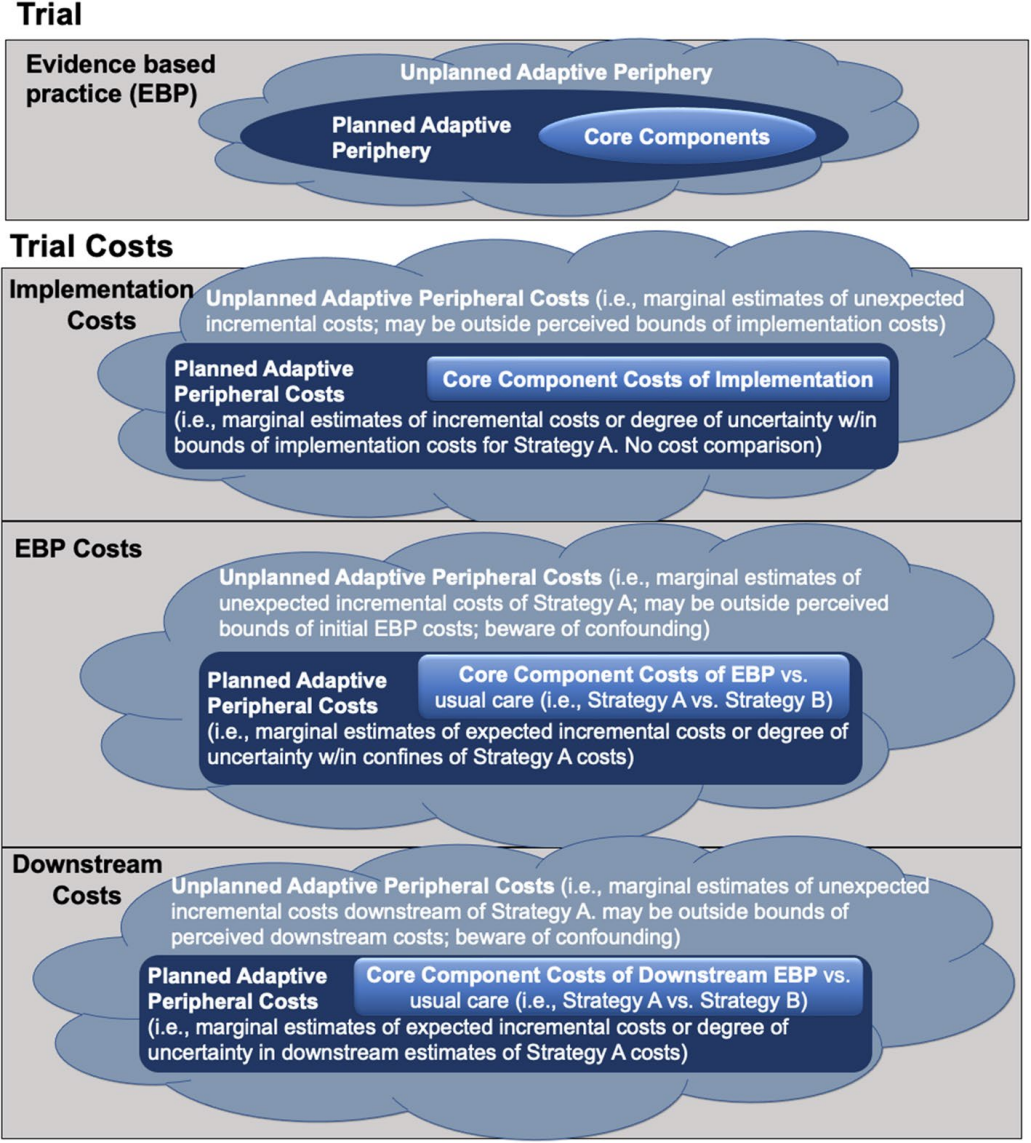
Core components, planned adaptive periphery, and unplanned adaptive periphery. The relationship is illustrated for both the trial components and the trial costs (i.e., implementation, intervention, and downstream)

Figure 1 conceptualizes adaptations to an EBP and its implementation that warrant economic evaluation. In the cases of the EBP, there are core components to consider, in addition to *planned* adaptive periphery, as well as *unplanned* adaptive periphery. This relationship is illustrated for both the trial components (i.e., EBP) and the trial costs (i.e., intervention costs, implementation costs, and downstream costs). Studies can be designed to monitor the impact of adaptations within the planned adaptive periphery. Economic evaluation can estimate the marginal costs for a particular implementation strategy, the marginal estimates of incremental costs of the EBP within the confines of the costs of a particular implementation strategy, and the marginal costs in downstream estimates of the implemented intervention. However, tracking downstream effects may be subject to confounding because it may not be clear what caused the effect.

Economic methods can also be used to estimate the marginal cost in the case of unplanned or reactive adaptation. Unplanned adaptation is the change to the content or strategy of EBP delivery that is not specified in the standard treatment protocol [23]. From an evaluation perspective, unplanned adaptations could be in response to unexpected contextual changes (e.g., changing the structure of a treatment protocol due to the onset of COVID-19) or could reflect a deficit of implementation fidelity (e.g., drifting from the protocol) and create noise that could limit the interpretation or reduce the statistical power of estimating the consequences of these adaptations. The economic consequences of unplanned adaptation decisions may be difficult to address comprehensively using standard evaluation methods. One issue is the problem of *endogeneity* that confounds all unplanned adaptations. Endogeneity means that the choice to adapt was based on internal decisions, as opposed to being randomly assigned. This prevents causal estimates because the adaptation is correlated with unobserved confounders. When estimating EBP costs, unplanned adaptive peripheral costs are the marginal estimates of unexpected incremental costs. For downstream costs, these are the marginal estimates of unexpected incremental costs downstream of implementation. In all cases, these estimates may be outside the perceived bounds of the initial EBP costs and confounding is possible.

**Fig. 2:**
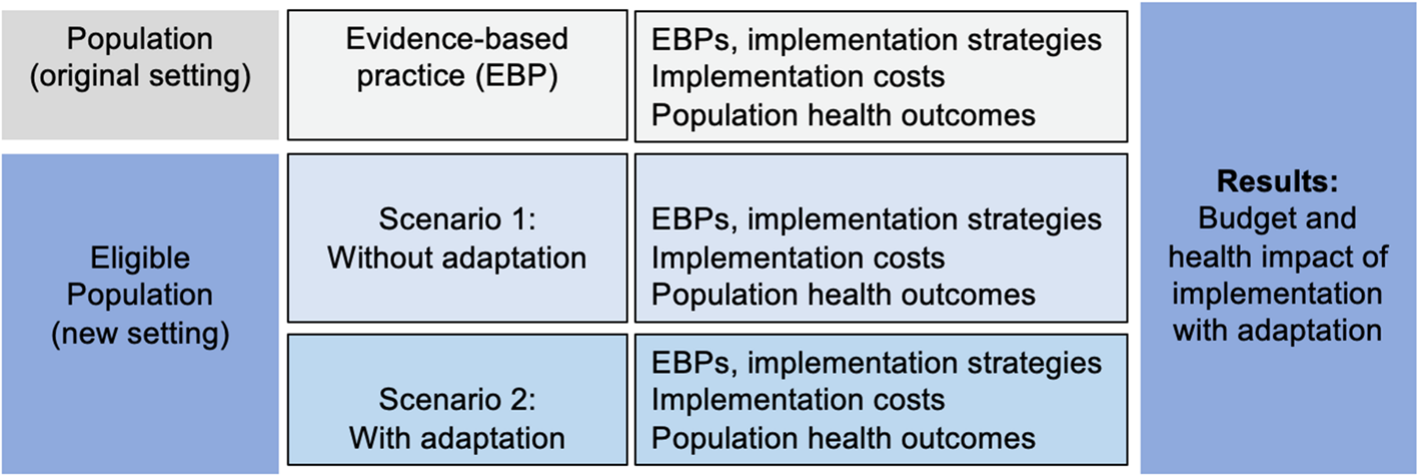
Model structure diagram for a budget impact analysis of implementation with adaptation (adapted from Mauskopf et al.) [24]

For a given EBP, estimates may exist from the original trial on the cost or cost-effectiveness of implementing the intervention. However, because an adapted EBP will rely on different local resources across various delivery settings, costs could differ substantially between contexts. When the EBP is implemented in a new setting, one scenario could involve implementing the EBP without adaptation. Although some costs might be lower in the new setting — for example, if the original trial required more developmental work for an implementation infrastructure — it is possible that the EBP will achieve suboptimal outcomes due to the limited fit between the EBP and the new delivery setting. Therefore, an alternative scenario involves adaption to improve the fit of the EBP in the new setting. In this scenario, there is an incremental cost to implementing the EBP beyond the costs involved in the original setting, and there is an incremental change in implementation outcomes beyond the scenario that does not include adaptation. This rationale for studying the economics of adaptation is applicable to both cost-effectiveness analysis and budget impact analysis and depends on whether a standard has been established by one or more trials evaluating the EBP. The model structure diagram for a budget impact analysis [24] of implementation with an adaptation scenario is provided in Fig. 2 for illustrative purposes, although a similar logic can be followed for addressing adaptations in a cost-effectiveness analysis. If the implementation of an EBP in a new setting without adaptation results in a loss in net benefits from the intervention, these models can examine the extent to which the adaptation restores these benefits.

#### Important considerations for the economics of adaptation

The analysis of implementation studies includes costs that accrue to or are paid by the decision-maker and other costs relevant to stakeholders [25]. Implementation stakeholders are often faced with the decision of whether the adaptation should be made, based on prior knowledge and future projections of the value of EBPs. Stakeholders include those who are making the adaptation decision (whether top-down or bottom-up), but also those who will receive the results of the economic evaluation, including internal and external stakeholders. Therefore, specifying the appropriate economic perspective helps in identifying how and why certain adaptations should be costed and is essential for the effective replication of EBP implementation or in planning for potential adaptation. Given that implementation stakeholders are often interested in short-term rather than longer term outcomes, we focus the discussion on the evaluation of the economic impact of adaptation on intervention and implementation costs at the expense of downstream costs, especially when adaptations are involved. This discussion is applicable to both cost-effectiveness analysis which evaluates whether an EBP provides value relative to an existing intervention and budget impact analysis which estimates the financial consequences of adopting a new EBP.

Another important consideration in the economic evaluation of implementation within the context of adaptation is to specify the time period of interest. In traditional economic evaluation, the time horizon is any period over which economic value could be expected to differ across the comparators. The traditional approach of selecting a fixed value time horizon may be inappropriate. Philips et al. discussed alternative approaches to the selection of time horizons for valuing implementation alternatives and the benefits of explicitly modeling future changes and advocate for the selection of a common time horizon after accounting for the effects of anticipated changes in short-term adaptations [26]. Timing of the adaptation may influence cost, and it is plausible to observe diminishing returns in cost-savings for introducing an adaptation later in the implementation process. Given the complexity of adaptation decisions, implementation science frameworks are needed to evaluate costs of adaptations.

### Case study: costing adaptations in implementation of opioid reassessment clinics

Implementation science frameworks can also clarify how adaptations could simultaneously affect primary target outcomes of an implementation study as well as its economic evaluation. Clarifying this a priori helps in planning for how to measure and report adaptations and make more appropriate cost estimates related to outcomes of interest. Table 2 illustrates a case study of adaptations in opioid reassessment clinic (ORC) [27] implementation, organized by the domains of the Reach, Effectiveness, Adoption, Implementation, and Maintenance (RE-AIM) framework [28]. For each RE-AIM outcome, we present an adaptation example, along with its cost relevance, considerations for cost analysis, and a comparison of the implications of planned and unplanned adaptation. In the case study, effectiveness is measured as the number of patients on high-risk long-term opioid therapy who are transitioned to safer regimens. A new initiative at the clinical site requires regular monitoring of patients transferred to safer regimens. Costing this adaptation involves measuring the time and resources required for monitoring patients after the initiative is launched. One method to consider here is interrupted time series that could examine the cost of adaptation on the effectiveness of the outcome per unit of time. However, in the case of unplanned adaptation, a causal inference may not be achieved due to endogeneity. One of the implementation outcome measures in the case study is the number of patients successfully completing treatment with the opioid reassessment clinic. The adaptation in this case involves hiring a new addiction specialist on site with specialty in tapering. Implications of the adaptation involve added salary for the new specialist, in addition to more time consulting and coordinating care with more patients. The additional implementation costs could be assessed in relevance to the increased rate of outcome per unit time. If this adaptation is planned, a causal inference may be achieved with random assignment of the adaptation.

**Table 2:** Example of costing adaptations organized by the RE-AIM framework

RE-AIM domain	Outcome	Adaptation	Cost relevance implications of adaptation	Considerations for cost analysis	Planned vs. unplanned
Reach	Number of patients with at least one ORC appointment	Expanding eligibility criteria for ORC services	Time/resources required for serving additional patients	Interrupted time series could examine cost of new policy on patient reach	Unplanned. Causal inference may not be achieved due to endogeneity
Effectiveness	Number of patients on high-risk LTOT transitioned to safer regimens	New initiative at site requires regular monitoring of patients transferred to safer regimens	Time/resources required for monitoring patients post launch of the initiative	Interrupted time series could examine cost of adaptation on effectiveness per time unit	Unplanned. Causal inference may not be achieved due to endogeneity
Adoption	Number of providers referring patients for opioid reassessment	New dashboard for PCPs to determine patients pre-requisites for ORC	Start-up costs for dashboard development along w/ training in the use of dashboard	The additional implementation costs could be assessed in relevance to outcome of interest	Unplanned. Causal inference may not be achieved due to endogeneity
Implementation	Number of patients seen by ORC	Tele-ORC tested at random sites due to COVID	Start-up costs for training on Tele-ORC and coordination. Changes in patient health care utilization due to tele-health delivery	Sensitivity analysis could show cost differentials in these delivery methods	Planned. Causal inference may be achieved with random assignment
	Number of patients successfully completing treatment with ORC	New addiction specialist hired on site w/ specialty in tapering	Salary addition for new specialist on the implementation team. More time consulting/coordinating care w/ more patients	The additional implementation costs could be assessed in relevance to increased rate of outcome per unit time	Unplanned. Causal inference may not be achieved due to endogeneity
Maintenance	Number of providers referring patients for opioid reassessment and completed consults, and number of patients seen	Tele-ORC maintained due to COVID and expanded in a step-wedged fashion to other sites	Additional start-up costs for training on Tele-ORC and coordination. Patient health care utilization continues to differ due to tele-health delivery	Sensitivity analysis could show cost differentials in these delivery methods	Planned. Causal inference may be achieved due to random assignment

*ORC* opioid reassessment clinic, *LTOT* long-term opioid treatment

### Economic approaches to costing adaptations

Adaptations occur across a continuum of implementation study designs, ranging from trials testing adapted versions of the EBP to studies that allow for local adaptations during the implementation process, with the potential for an infinite number of versions of the original EBP. Herein, we discuss various economic approaches to costing adaptations, namely micro-costing, time-based activity costing, minimal intervention needed for change, and simulation modeling.

#### Micro-costing approaches

At one end of this continuum, micro-costing approaches have been applied in research that integrates adaptation of EBPs and implementation strategies at the planning stage using innovative study designs, such as the multiphase optimization strategy trial (MOST) design [29]. An adaptive implementation strategy can consist of several discrete strategies delivered in sequence and in different combinations. In adaptive implementation strategies, the type and dose of implementation strategy delivered to a site may be tailored in direct response to levels of adoption of EBPs observed among specific sites. Tailoring of implementation strategies could be based on circumstances that may not be observable at baseline. In one example, Collins and colleagues evaluated multiple intervention components for a smoking cessation trial with a MOST design [30]. The study used a phase-based approach to guide the choice of intervention components and outcome measures through randomized experimentation, while using the MOST framework to ensure that the intervention was not only effective, but also efficient and scalable. Matching implementation strategies to clinical context can be viewed as an optimization problem, whereby the objective is to determine the most cost-effective approach to achieve the desired health outcome.

#### The sequential multiple assignment randomized trial

One type of MOST used in optimizing decision rules of a time-varying adaptive intervention is the sequential multiple assignment randomized trial (SMART). A cluster randomized trial of community-based clinics with a SMART design evaluated a standard versus enhanced implementation strategy to improve outcomes of an EBP to treat mood disorders [31]. The trial evaluated implementation strategies of varying intensity, with a low-intensity strategy that includes EBP packaging, training, and technical assistance, followed by a medium-intensity strategy involving facilitation by external experts, and a high-intensity strategy involving internal facilitation with protected time for internal staff to support EBP implementation. Implementation strategies were evaluated for their cost-effectiveness through random assignment in three stages, whereby sites not responding to the implementation strategy in the previous stage were further randomized to receive a supplemental implementation strategy in the subsequent stage [22]. Findings suggested that the most cost-effective implementation support starts with a less intensive, less costly implementation strategy and increases as needed to enhance EBP uptake [22]. A SMART design is also being applied to the implementation of clinical guidelines for opioid prescribing in primary care settings, in which clinics are randomized to receive a sequence of implementation strategies that address implementation concerns at the health system, clinic, and provider levels [32]. This ongoing study aims to identify the most cost-effective sequence and combination of implementation strategies [32].

#### Activity-based costing approaches

Calculation of the expected value of specific implementation strategies and their adaptations requires determining the change in implementation levels and estimating the value of those changes. Comparing the value of such changes against their costs allows for the estimation of the expected value of the implementation strategies and adaptations. Empirical studies using micro-costing methods such as activity-based costing can inform decision-makers about the opportunity costs of alternative health care interventions or strategies and how they change over time [33]. This involves tracking the inputs used to produce the EBP and multiplying the quantity of inputs by their input costs. The underlying assumption behind these methods is that the adaptation to improve implementation of the intervention results in a net health benefit from the intervention being tested. Such a gain in net health benefit is then compared with the cost of the implementation strategy to quantify whether it is an appropriate use of health care resources by estimating the value of implementation for a defined patient population and health care budget. Although relying on routinely generated data sources for analysis, such as electronic health record data, is preferrable due to its low burden, primary data collection may be necessary for measures that are not typically captured in existing data sources.

**Fig. 3:**
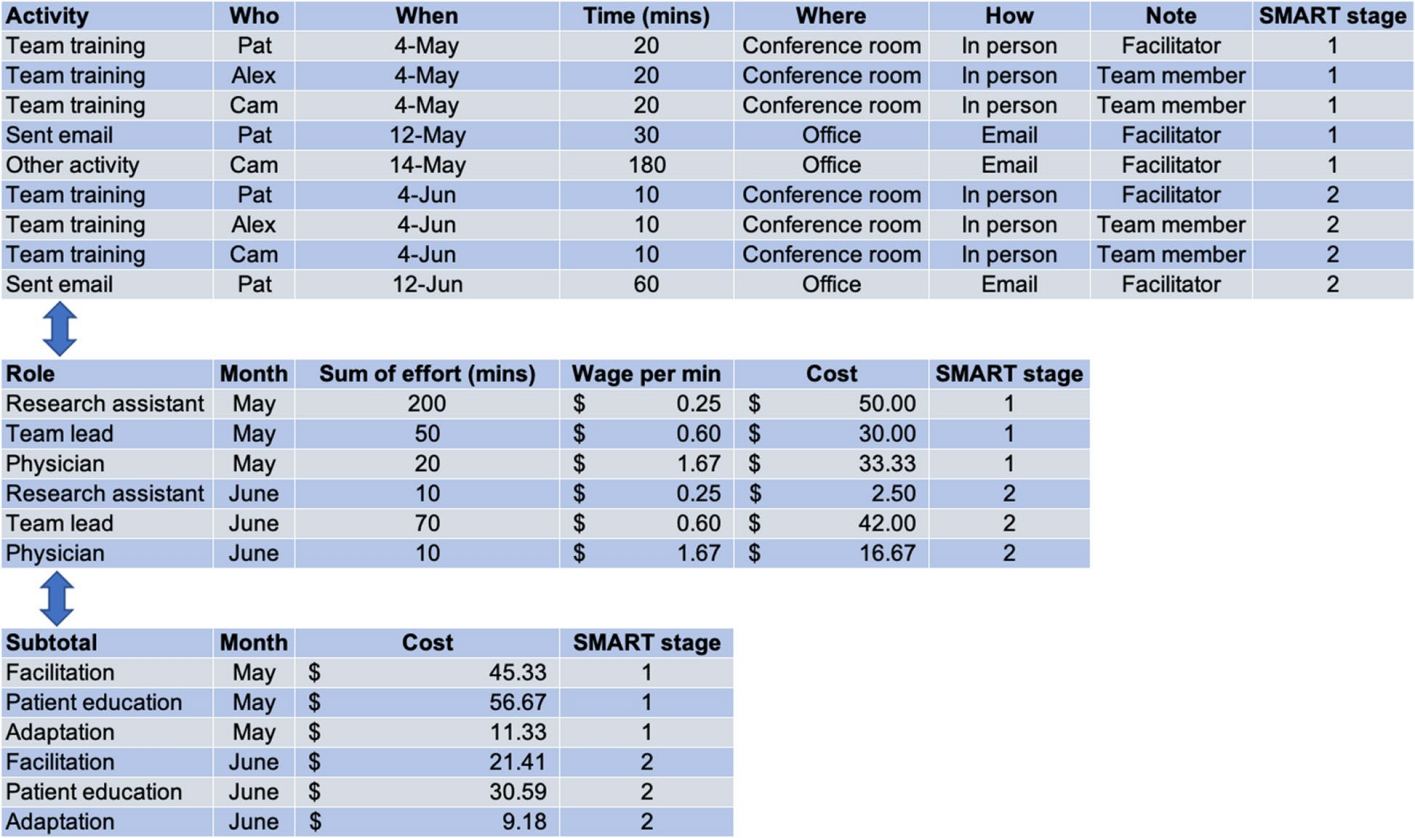
Measuring input costs to understand the effect of adaptation

The example provided in Fig. 3 outlines one method of measuring costs (while considering adaptations) with activity-based costing, but there are other types of micro- and macro-costing methods (e.g., cost-adjusted charges or total reimbursement, and gross-costing) that are discussed elsewhere [34]. Figure 3 illustrates an approach for the measurement of input labor costs with the purpose of understanding the impact of adaptation on such costs. Although not included in this example for simplicity, other input costs (e.g., supplies, technology) may be considered as well. This hypothetical example considers the case of a planned adaptation within the context of a SMART design, whereby participants in one of the trial arms underwent a planned adaptation. Considering an activity-based costing dataset that is derived from a cost survey or log (panel A), the information is then summarized into an intermediate dataset with computed costs (panel B). In this stage, decision-makers will need to address potentially missing data from the activity logs. For example, the analysis should be informed by a plausible assumption for the mechanism behind the missing data (i.e., whether the likelihood that the data are missing is independent of or dependent upon the observed or unobserved values) [35]. The intermediate dataset is then transformed into a final analytic dataset for analysis, in which the costs attributable to the adaptation are derived as a distinct category based on the relevant activities (panel C). The variable costs in the adapted approach can be used to estimate the impact of the adaptation on health or system outcomes in determining the marginal benefit (or harm) based on the adaptation. In line with the concept of unplanned adaptations, this approach could also capture unplanned adaptations, by continuing to monitor input costs as local modifications arise. This approach could also be applied to retrospective studies, subject to the availability of input data. With retrospective data, it may be very difficult to use micro-costing if it is unclear when the adaptation took place and the effort that was expended before and after the adaptation. However, if retrospective micro-costing data already exist for an adaptation at a given site, these data may be invaluable when estimating costs to expand an adaptation to new sites and can be used as a tactic when approaching decision-makers.

#### Minimal intervention needed for change (MINC) approach

One approach that has been recently introduced to optimize the delivery of EBPs is the concept of “minimal intervention needed for change” or MINC [36]. The goal of MINC is to provide a minimum standard for comparing low- vs. high-intensity EBPs to evaluate the relative improvements based on their relative costs. One question is as follows: how much more (or less) will the EBP cost with the adaptations and how do these changes influence the cost of EBP delivery or the implementation strategy? Not all adaptations are costly. Some adaptations involve recurring expenditures as opposed to new expenditures. Defining the core elements vs. the adaptable components of an EBP or implementation strategy is critical for the downstream economic evaluation of EBP delivery under different adaptation scenarios. Although MINC is a promising approach, its premise relies on introducing another comparison condition (i.e., the less intensive, less costly condition) that may or may not align with the required adaptation.

#### Simulation modeling approaches

In addition to economic evaluation based on empirical evidence, simulation modeling can be used to overcome limited follow-up in implementation studies. Common dynamic simulation modeling methods to inform the implementation of EBPs in health care systems include system dynamics, discrete event simulation, and agent-based modeling [37]. These approaches to dynamic systems allow for considering the complexity of the system by modeling the upstream and downstream economic consequences of adaptations in complex health care systems. Simulation modeling enables decision-makers to evaluate scenarios outside of the observed norm, extend the time of observation, and strengthen and relax assumptions, which is typically not feasible in the real world. While it is inherently challenging to simulate downstream cost estimates over the long-term, overlaying simulations with probabilistic models of plausible health care disruptions (e.g., varying degrees of pandemic-related impacts) may provide more confidence in the standard error around the estimate.

Although there are distinct advantages of simulation modeling over other economic evaluation models, these approaches can introduce challenges with respect to both analysis and interpretation. For instance, simulation models require extensive data that may not be available in the detail required by the model, especially when considering unintended economic consequences of an adaptation. However, although simulation models might require detailed or unavailable data, they are also able to account for this limitation in a way that other modeling approaches are unable to. Simulation modeling can also present challenges for decision-makers — i.e., end users of such models. Given the additional complexity of such models, decision-makers may find them more difficult to comprehend, assess their validity, and interpret their findings. Although simulation modeling can be intimidating for some decision-makers, there is a strong evidence base to make such methods more accessible — e.g., through group model building and community-based system dynamics [38, 39]. These dynamic approaches allow for the costs and effects of the implementation strategy to improve implementation of an EBP across multiple time periods, whereas the traditional static approach assumes that the impact occurs entirely in a single time period. Varying marginal costs and benefits can be integrated into this approach by allowing for varying costs and benefits for the value of the EBP and of implementation strategies. Finally, even if simulations are not able to generate a precise point estimate, the simulations can generate a range of costs, and the bounds may provide sufficient information for decision-makers [40].

#### Coincidence analysis as an alternative to simulation modeling

One alternative method that has recently emerged to address the real-world complexity of dynamic implementation is coincidence analysis [41]. In coincidence analysis, researchers can apply an algorithm specifically designed for causal inference across entire datasets to identify specific combinations of components and conditions that consistently lead to improved outcomes, regardless of sample size [41]. However, one challenge with using coincidence analysis is that it requires the measures to be temporally aligned with the outcome, whereas downstream costs, by definition, are not temporally connected. Therefore, coincidence analysis may be useful to measure the costs of implementation strategies when the method used has sufficient precision to do so.

### Addressing uncertainty and other considerations for costing adaptations

The results of any economic analysis contain inherent uncertainty due to the estimated nature of current inputs, other assumptions (e.g., the same observed benefits extend beyond the observation period), and predictions about the future. This uncertainty in economic modeling is exacerbated in the case of adaptation. Therefore, it is important for economic analysis of adaptation to include both scenario and sensitivity analyses [42]. The first source of uncertainty is related to the measures and assumptions that vary from the setting of the original trial as the intervention is implemented within a new site. Examples of measures that vary by setting include population characteristics, health system treatment patterns, and the disease prevalence within the health system. Scenario analysis is useful for this source of uncertainty. Local information should be used to inform alternative plausible scenarios by varying the value of these input measures. However, caution should be exercised to avoid the excessive use of assumptions rather than data and the inadequate characterization of uncertainty [43]. The second source of uncertainty is for the measures and other assumptions that are estimated with uncertainty regardless of the setting. This uncertainty could be due to changes in measures over the analysis time horizon and unpredictable future events. Sensitivity analysis is useful for this source of uncertainty. To the extent possible, the ranges of alternative values used for the sensitivity analysis should be based on observed variability in each measure, and information should be provided on how these ranges were derived. For example, the ranges used for effectiveness could be the 95% confidence limits from the original trial. For uncertain variables that lack observed data, expert opinion on likely ranges may be needed. When presenting results of the economic analysis, the base-case analysis results using single or default values for both the inputs known and unaffected by the adaptation and those for which there is uncertainty. Then, the alternative sets of results should be presented by changing the uncertain input measure one at a time or by changing a group of parameter values.

#### New estimation approaches are needed

One area where improved methods are needed is in estimating the economic consequences of the adaptation, compared with no adaptation, based on individual components of the EBP or implementation strategy. Using current micro-costing approaches, one would start with an estimate of the current EBP components without the adaptation under the original setting. However, producing projections of change in the effectiveness of various components once the adaptation is introduced would be based on assumptions or best-guess estimates. An additional assumption that is often made is that implementation capacity is equal across sites. Some adopting sites that have greater implementation capacity may be able to adapt more quickly or achieve superior outcomes. Guidance for methods to estimate the effect of adaptation on EBP or implementation strategy components would be useful. In addition to FRAME, FRAME-IS is a recent extension of the tool that can be used for documenting adaptations to implementation strategies [44].

## Conclusions

Economic evaluation of adaptations to EBP delivery is complex due to the multifaceted interactions involved, including various stakeholders, health care organizations, implementation processes, and technological innovations. Considering these challenges, researchers should strive to engage stakeholders in understanding the circumstances leading to the adaptation and its implications. As with any economic evaluation, clearly defining the problem and considering the appropriate perspective and time horizon are critical to guiding the purpose and scope of the evaluation. Mixed methods research guided by implementation science frameworks is important to establish the circumstances surrounding the adaptions and data availability. Next, researchers should consider the appropriateness of simulation modeling over traditional models for economic evaluation. Recently, the Professional Society for Health Economics and Outcomes Research (lSPOR) proposed a checklist to assist researchers and decision-makers in deciding whether simulation methods are appropriate to address specific health system problems [37]. The checklist can be used by health care delivery and implementation researchers, as well as decision-makers, in planning system interventions that address complex challenges in delivering effective and efficient care. Recent developments in implementation science and data science present opportunities to broaden and deepen our scientific understanding of the economics of adaptation. Beyond simulation modeling, additional checklists and reporting templates are available from ISPOR and other international standards organizations for applications in health economics and outcomes research to inform health care decision-making [45, 46].

The National Academy of Medicine has prioritized a paradigm shift towards a learning health care system, characterized by continuous learning and quality improvement with continuity in clinical data collection informing faster and more iterative adaptations [47]. This dynamic approach to evidence development and application integrates adaptative, contextually sensitive continuous quality improvement with the challenge of EBP sustainment [5]. Although a significant gap remains in achieving the goals of a learning health care system, progress will only be made if health systems can adapt to their evolving environments and the economic case for such adaptations can be demonstrated. Specifically, it is crucial to understand the differences between costing at baseline and real-world delivery. Further research on the economic implications of adaptations during the implementation process is necessary to improve EBP scaleup and sustainability.

## Data Availability

This paper does not involve the use of specific data or materials.

## Abbreviations

EBP: Evidence-based practice
FRAME: Framework for Reporting Adaptations and Modifications to Evidence-based interventions
MINC: Minimal intervention needed for change
MOST: Multiphase optimization strategy trial
SMART: Sequential multiple assignment randomized trial

## References

[1] Owens DK, Qaseem A, Chou R, Shekelle P (2011). Clinical Guidelines Committee of the American College of P: High-value, cost-conscious health care: concepts for clinicians to evaluate the benefits, harms, and costs of medical interventions. Ann Intern Med.

[2] Roberts SLE, Healey A, Sevdalis N (2019). Use of health economic evaluation in the implementation and improvement science fields-a systematic literature review. Implement Sci.

[3] Gold HT, McDermott C, Hoomans T, Wagner TH (2022). Cost data in implementation science: categories and approaches to costing. Implement Sci.

[4] Glasgow RE, Battaglia C, McCreight M, Ayele RA, Rabin BA (2020). Making implementation science more rapid: use of the RE-AIM framework for mid-course adaptations across five health services research projects in the Veterans Health Administration. Front Public Health.

[5] Chambers DA, Glasgow RE, Stange KC (2013). The dynamic sustainability framework: addressing the paradox of sustainment amid ongoing change. Implement Sci.

[6] Stirman SW, Miller CJ, Toder K, Calloway A (2013). Development of a framework and coding system for modifications and adaptations of evidence-based interventions. Implement Sci.

[7] Escoffery C, Lebow-Skelley E, Haardoerfer R, Boing E, Udelson H, Wood R, Hartman M, Fernandez ME, Mullen PD (2018). A systematic review of adaptations of evidence-based public health interventions globally. Implement Sci.

[8] Evans RE, Moore G, Movsisyan A, Rehfuess E, Panel A (2021). Arnold APcoL: How can we adapt complex population health interventions for new contexts? Progressing debates and research priorities. J Epidemiol Community Health.

[9] Aarons GA, Green AE, Palinkas LA, Self-Brown S, Whitaker DJ, Lutzker JR, Silovsky JF, Hecht DB, Chaffin MJ (2012). Dynamic adaptation process to implement an evidence-based child maltreatment intervention. Implement Sci.

[10] Rogers E (2003). Diffusion of innovations fifth.

[11] Charters WW, Pellegrin RJ. Barriers to the innovative process: Four case studies of differentiated staffing. Educ Admin Q. 1973;9(1).

[12] Leviton L, Henry B (2011). Better information for generalizable knowledge: systematic study of local adaptation. American Public Health Association 139th Annual Meeting and Exposition.

[13] Chambers DA, Norton WE (2016). The adaptome: advancing the science of intervention adaptation. Am J Prev Med.

[14] Sculpher M (2000). Evaluating the cost-effectiveness of interventions designed to increase the utilization of evidence-based guidelines. Fam Pract.

[15] Mason J, Freemantle N, Nazareth I, Eccles M, Haines A, Drummond M (2001). When is it cost-effective to change the behavior of health professionals?. JAMA.

[16] Whyte S, Dixon S, Faria R, Walker S, Palmer S, Sculpher M, Radford S (2016). Estimating the cost-effectiveness of implementation: is sufficient evidence available?. Value Health.

[17] Mewes JC, Steuten LMG (2017). C IJ, MJ IJ, van Harten WH: Value of implementation of strategies to increase the adherence of health professionals and cancer survivors to guideline-based physical exercise. Value Health.

[18] Wiltsey Stirman S, Baumann AA, Miller CJ (2019). The FRAME: an expanded framework for reporting adaptations and modifications to evidence-based interventions. Implement Sci.

[19] Scheirer MA (2013). Linking sustainability research to intervention types. Am J Public Health.

[20] Moore G, Campbell M, Copeland L, Craig P, Movsisyan A, Hoddinott P, Littlecott H, O'Cathain A, Pfadenhauer L, Rehfuess E (2021). Adapting interventions to new contexts-the ADAPT guidance. BMJ.

[21] Dopp AR, Mundey P, Beasley LO, Silovsky JF, Eisenberg D. Mixed-method approaches to strengthen economic evaluations in implementation research. Implement Sci. 2019:14. 10.1186/s13012-018-0850-6 PMC6329154 30635001

[22] Eisman AB, Hutton DW, Prosser LA, Smith SN, Kilbourne AM (2020). Cost-effectiveness of the Adaptive Implementation of Effective Programs Trial (ADEPT): approaches to adopting implementation strategies. Implement Sci.

[23] Wiltsey Stirman S, Gutner CA, Crits-Christoph P, Edmunds J, Evans AC, Beidas RS (2015). Relationships between clinician-level attributes and fidelity-consistent and fidelity-inconsistent modifications to an evidence-based psychotherapy. Implement Sci.

[24] Mauskopf JA, Sullivan SD, Annemans L, Caro J, Mullins CD, Nuijten M, Orlewska E, Watkins J, Trueman P (2007). Principles of good practice for budget impact analysis: report of the ISPOR Task Force on good research practices--budget impact analysis. Value Health.

[25] Jones Rhodes WC, Ritzwoller DP, Glasgow RE (2018). Stakeholder perspectives on costs and resource expenditures: tools for addressing economic issues most relevant to patients, providers, and clinics. Transl Behav Med.

[26] Philips Z, Claxton K, Palmer S (2008). The half-life of truth: what are appropriate time horizons for research decisions?. Med Decis Making.

[27] Becker WC, Edmond SN, Cervone DJ, Manhapra A, Sellinger JJ, Moore BA, Edens EL (2018). Evaluation of an integrated, multidisciplinary program to address unsafe use of opioids prescribed for pain. Pain Med.

[28] Glasgow RE, Vogt TM, Boles SM (1999). Evaluating the public health impact of health promotion interventions: the RE-AIM framework. Am J Public Health.

[29] Collins LM, Murphy SA, Strecher V (2007). The multiphase optimization strategy (MOST) and the sequential multiple assignment randomized trial (SMART): new methods for more potent eHealth interventions. Am J Prev Med.

[30] Collins LM, Baker TB, Mermelstein RJ, Piper ME, Jorenby DE, Smith SS, Christiansen BA, Schlam TR, Cook JW, Fiore MC (2011). The multiphase optimization strategy for engineering effective tobacco use interventions. Ann Behav Med.

[31] Kilbourne AM, Almirall D, Eisenberg D, Waxmonsky J, Goodrich DE, Fortney JC, Kirchner JE, Solberg LI, Main D, Bauer MS (2014). Protocol: Adaptive Implementation of Effective Programs Trial (ADEPT): cluster randomized SMART trial comparing a standard versus enhanced implementation strategy to improve outcomes of a mood disorders program. Implement Sci.

[32] Quanbeck A, Almirall D, Jacobson N, Brown RT, Landeck JK, Madden L, Cohen A, Deyo BMF, Robinson J, Johnson RA (2020). The Balanced Opioid Initiative: protocol for a clustered, sequential, multiple-assignment randomized trial to construct an adaptive implementation strategy to improve guideline-concordant opioid prescribing in primary care. Implement Sci.

[33] Wagner TH (2020). Rethinking how we measure costs in implementation research. J Gen Intern Med.

[34] Lipscomb J, Yabroff KR, Brown ML, Lawrence W, Barnett PG (2009). Health care costing: data, methods, current applications. Med Care.

[35] Faria R, Gomes M, Epstein D, White IR (2014). A guide to handling missing data in cost-effectiveness analysis conducted within randomised controlled trials. Pharmacoeconomics.

[36] Glasgow RE, Fisher L, Strycker LA, Hessler D, Toobert DJ, King DK, Jacobs T (2014). Minimal intervention needed for change: definition, use, and value for improving health and health research. Transl Behav Med.

[37] Marshall DA, Burgos-Liz L (2015). MJ IJ, Osgood ND, Padula WV, Higashi MK, Wong PK, Pasupathy KS, Crown W: Applying dynamic simulation modeling methods in health care delivery research-the SIMULATE checklist: report of the ISPOR simulation modeling emerging good practices task force. Value Health.

[38] Hovmand PS (2014). Group model building and community-based system dynamics process. *Community based system dynamics*.

[39] Gerritsen S, Harre S, Rees D, Renker-Darby A, Bartos AE, Waterlander WE, et al. Community group model building as a method for engaging participants and mobilising action in public health. Int J Environ Res Public Health. 2020;17(10). 10.3390/ijerph17103457 PMC7277214 32429183

[40] Mullahy J, Venkataramani A, Millimet DL, Manski CF (2021). Embracing uncertainty: the value of partial identification in public health and clinical research. Am J Prev Med.

[41] Whitaker RG, Sperber N, Baumgartner M, Thiem A, Cragun D, Damschroder L, Miech EJ, Slade A, Birken S (2020). Coincidence analysis: a new method for causal inference in implementation science. Implement Sci.

[42] Sullivan SD, Mauskopf JA, Augustovski F, Jaime Caro J, Lee KM, Minchin M, Orlewska E, Penna P, Rodriguez Barrios JM, Shau WY (2014). Budget impact analysis-principles of good practice: report of the ISPOR 2012 Budget Impact Analysis Good Practice II Task Force. Value Health.

[43] Drummond M, Sculpher M (2005). Common methodological flaws in economic evaluations. Med Care.

[44] Miller CJ, Barnett ML, Baumann AA, Gutner CA, Wiltsey-Stirman S (2021). The FRAME-IS: a framework for documenting modifications to implementation strategies in healthcare. Implement Sci.

[45] Society for Medical Decision Making. Modeling Good Research Practices Task Force [https://smdm.org/hub/page/modeling-good-research-practices-task-force/publications]

[46] ISPOR. Good practices reports & more [https://www.ispor.org/heor-resources/good-practices]

[47] Olsen L, Aisner D, McGinnis JM. Institute of Medicine (US). Roundtable on Evidence-Based Medicine. The learning healthcare system: workshop summary. Washington National Academies Pr. 2007. 21452449

